# Resolving Phenotypic Variability in Mitochondrial Diseases: Preliminary Findings of a Proteomic Approach

**DOI:** 10.3390/ijms251910731

**Published:** 2024-10-05

**Authors:** Michela Cicchinelli, Guido Primiano, Serenella Servidei, Michelangelo Ardito, Anna Percio, Andrea Urbani, Federica Iavarone

**Affiliations:** 1 Dipartimento di Scienze Biotecnologiche di Base, Cliniche Intensivologiche e Perioperatorie, Università Cattolica del Sacro Cuore, 00168 Rome, Italy; michela.cicchinelli@unicatt.it (M.C.); anna.percio@unicatt.it (A.P.); andrea.urbani@unicatt.it (A.U.); federica.iavarone@unicatt.it (F.I.); 2 Dipartimento di Neuroscienze, Università Cattolica del Sacro Cuore, 00168 Rome, Italy; serenella.servidei@unicatt.it (S.S.); 19.miche.97@gmail.com (M.A.); 3 Fondazione Policlinico Universitario “Agostino Gemelli” IRCCS, 00168 Rome, Italy

**Keywords:** mitochondria, mitochondrial diseases, PEO, proteomics, omics technologies

## Abstract

The introduction of new sequencing approaches into clinical practice has radically changed the diagnostic approach to mitochondrial diseases, significantly improving the molecular definition rate in this group of neurogenetic disorders. At the same time, there have been no equal successes in the area of in-depth understanding of disease mechanisms and few innovative therapeutic approaches have been proposed recently. In this regard, the identification of the molecular basis of phenotypic variability in primary mitochondrial disorders represents a key aspect for deciphering disease mechanisms with important therapeutic implications. In this study, we present data from proteomic investigations in two subjects affected by mitochondrial disease characterized by a different clinical severity and associated with the same variant in the *TWNK* gene, encoding the mitochondrial DNA and RNA helicase with a specific role in the mtDNA replisome. Heterozygous pathogenic variants in this gene are associated with progressive external ophthalmoplegia and ptosis, usually with adult onset. The overall results suggest an imbalance in glucose metabolism and ROS production/regulation, with possible consequences on the phenotypic manifestations of the enrolled subjects. Although the data will need to be validated in a large cohort, proteomic investigations have proven to be a valid approach for a deep understanding of these neurometabolic disorders.

## 1. Introduction

Progressive External Ophthalmoplegia (PEO) is the most common primary mitochondrial myopathy (PMM), resulting from a single large de novo deletion of the mitochondrial DNA (mtDNA), inherited through autosomal dominant, autosomal recessive, or maternal transmission [1]. In particular, PMM is a specific group of genetic mitochondrial disorders with variable clinical features and characterized by oxidative phosphorylation (OxPhos) defects that primarily, but not exclusively, affect skeletal muscle [1]. These neurometabolic disorders are associated with variants in either nuclear or mitochondrial genes encoding proteins essential to mitochondrial function [2,3].

Over the years, an increasing number of genes involved in mtDNA replication and maintenance have been reported in patients with PMM in association with mtDNA multiple deletions or depletion (*ABAT*, *DGUOK*, *DNA2*, *MGME1*, *MPV17*, *POLG*, *POLG2*, *RNASEH1*, *RRM2B*, *SAMHD1*, *SLC25A4*, *SSBP1*, *SUCLA2*, *SUCLG1*, *TFAM*, *TK2*, *TOP3A*, *TWNK*, *TYMP, LIG3*) [4]. The adult-onset PEO syndrome is among those frequently caused by mtDNA-maintenance defects. Specifically, some of the main genetic defects responsible for the onset of this specific mitochondrial phenotype are variants in the nuclear *TWNK* gene, which encodes the mitochondrial DNA and RNA helicase with a key role in the mtDNA replisome. This specific helicase is part of the SF4 superfamily and the only replicative helicase required for mtDNA maintenance. Additional evidence suggests that Twinkle helicase plays a role in the regulation of mtDNA replication/maintenance and repair [5]. Overall, its functions are essential to maintain the functionality of mitochondrial processes [6]. Variants in the *TWNK* gene and consequent mtDNA replication defects can ultimately lead to mitochondrial dysfunction [7]. The direct consequence is a deficiency in energy production through the electron transport chain due to impaired respiratory function. At present, it is very difficult or impossible to systematically correlate the specific genetic variants with a specific phenotypic spectrum. For this reason, a case-by-case analysis is necessary for each PEO-associated variant.

Considering the mitochondrial involvement in several biological pathways, including the Krebs cycle and beta-oxidation of fatty acids, the dysfunction can influence the synthesis and metabolism of their key molecules and regulators, ultimately causing a significant metabolic remodeling [8]. In this context, the pathological increase of reactive oxygen species (ROS) plays a key role [9], capable of damaging cellular structures including mitochondria themselves and triggering inflammatory and apoptotic processes. Furthermore, regulatory pathways activate specific nuclear genes and metabolic processes in response to mitochondrial stress via mitochondrial retrograde signals [10].

The genetic defects causing primary mitochondrial disorders (PMD), such as PEO, can significantly influence not only the metabolic pathways directly connected with this organelle but also have repercussions on a wide range of cellular processes. These events play an important role in the pathogenesis of the disease and the severity of the clinical symptoms.

However, despite advances in preclinical research in this field and the significant improvements in genetic diagnostic approaches [11], a deep understanding of the pathological mechanisms of PMM has not yet been reached, such as for PEO syndrome [12,13]. Genetic and clinical complexity, therefore, represents a distinctive feature of PMD and reflects an equally complex molecular and metabolic mechanism underlying it, resulting in extreme difficulty in identifying effective therapeutic approaches [1,13].

Considering this scenario, proteomic analysis represents a valid strategy capable of providing detailed information on the protein content and changes that occur in a pathological condition [14], including PMM.

Here, we report the evidence obtained by analyzing the urinary proteome of two sisters affected by PEO, characterized by a different clinical severity and both carrying the c.1075G>A (p.Ala359Thr) variant in the *TWNK* gene.

Although skeletal muscle represents in many respects the tissue of choice to analyze for mitochondrial dysfunction, in this study, we preferred to investigate the urine of the involved patients for the following reasons: samples are easily available via non-invasive collection methods and in adequate quantities; the data obtained reflect the systemic remodeling of multi-organ disorders, such as PMM; and the samples are easily analyzed at different times of the natural evolution of the disease [15].

This study highlights the involvement of redox and glycolytic enzymes in the urine proteome of PEO patients, suggesting an oxidative stress-mediated regulation of glucose metabolism and redox processes. Additionally, the significant variation in enzyme expression between the two patients suggests a correlation with their phenotypic differences. 

## 2. Results

### 2.1. Patients

P(+) was a 62-year-old woman with progressive external ophthalmoplegia, bilateral ptosis, upper and lower limb girdle weakness, and exercise intolerance for more than 20 years. She also reported dysphagia for liquids, gastrointestinal dysmotility, balance instability, and headache. The histopathologic assessment of the patient’s deltoid muscle biopsy, performed at the age of 53 years, showed typical PMM findings characterized by mitochondrial proliferation with numerous classic ragged-blue fibers, which appeared pale with cytochrome c oxidase. The overall Newcastle Mitochondrial Disease Scale for Adults (NMDAS) [16] score was 31.

P(−), the older sister aged 63 years, presented only with mild bilateral ptosis and mild exercise intolerance (NMDAS 6). A muscle biopsy, performed at age 57, showed few ragged-blue and COX-negative fibers.

Both subjects carried c.1075G>A (p.Ala359Thr) variant in the *TWNK* gene.

### 2.2. Proteomic and Bioinformatic Analysis

**Figure 1:**
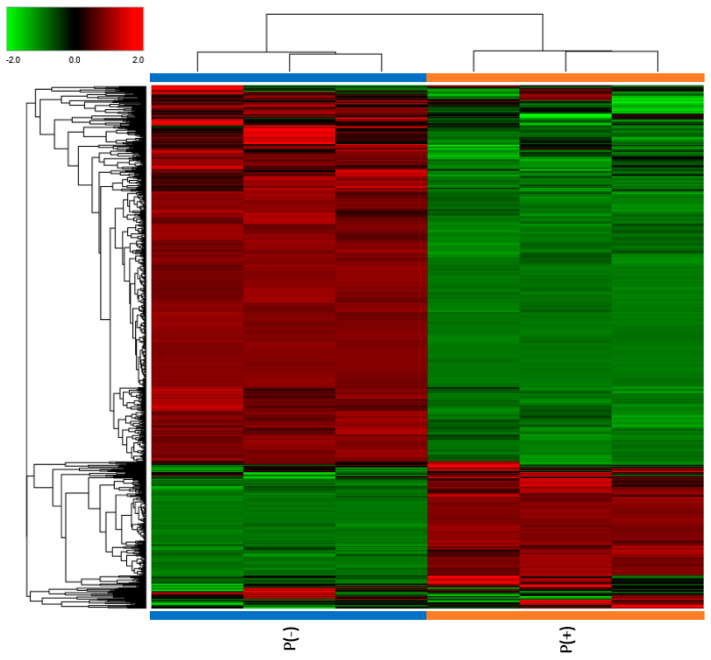
Clustergram illustrating proteomic analysis results. The protein abundances for each sample are presented in analytical triplicate. The values were previously treated by logarithmic transformation. Colors show the relative abundance in the two patients, with red and green indicating, respectively, higher and lower abundance.

Urine samples from the two patients and two healthy controls matched for sex and age were collected and analyzed through a mass spectrometry-based proteomic approach, used to identify and quantify the proteins. Noticeable differences in protein abundances were observed in the two patients, as evident from the Clustergram in Figure 1.

**Figure 2:**
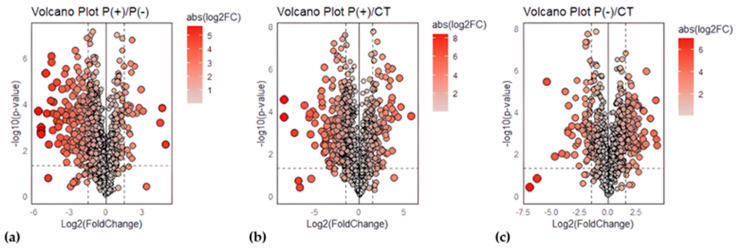
Volcano plots for urinary protein ratios. Volcano plots were constructed with the FC of protein abundances and the *p*-value, calculated by performing a two-paired t-test. The means of the triplicate abundances for the following ratios were compared: (**a**) P(+)/P(−) ratio, (**b**) P(+)/Ctrl ratio, (**c**) P(−)/Ctrl ratio. Red circles to the left and right of the FC threshold lines show proteins that have, respectively, significant decreases or increases in one sample compared to the other. The size and the red saturation of the circles increase proportionally to the degree of FC. The image was created using R (version 4.3.3., R Foundation for Statistical Computing, Vienna, Austria) software.

To verify our data, the results obtained from the proteomic analysis were compared between the two patients and against the healthy controls (Ctrl) through differential expression analysis, where the Fold Change (FC) of protein abundances in samples and the associated *p*-value were calculated. The analytical triplicate average abundances of proteins quantified in every sample were considered for the analysis and proteins that met the criteria of a FC greater than |1.5|, and a *p*-value less than 0.05 were considered statistically significant. Volcano plots showing minus log10 *p*-values and log2FC for the patient/patient ratio and patient/control ratio are reported in Figure 2.

An ontological analysis was conducted by investigating biological processes and pathways activated by differentially expressed proteins (FC > |1.5| and *p*-value < 0.05) through the Panther Classification System’s statistical overrepresentation test (Reference List: *Homo sapiens*, Test Type: Fisher’s Exact, Correction: FDR < 0.05). The analysis results were hierarchically sorted to highlight the relationships between enriched functional classes. All related classes within an ontology were clustered and interpreted as a group. Within each group, processes were sorted by descending Fold Enrichment and ascending FDR.

Specifically, the following differentially expressed proteins were investigated:

- Proteins identified in both patients that are downregulated in P(+)/Ctrl and upregulated in P(−)/Ctrl.
- Proteins identified in both patients that are upregulated in P(+)/Ctrl and downregulated in P(−)/Ctrl.
- Proteins commonly downregulated in both patients compared to controls.
- Proteins commonly upregulated in both patients compared to controls.

A total of 40 proteins identified in both patients were found to be downregulated in P(+) and upregulated in P(−). Among the biological processes, the most specific subclass was the carbohydrate catabolic process (FDR 3.93 × 10^−2^), which includes the chemical reactions and metabolic pathways involved in the breakdown of carbohydrate derivatives, such as glycolysis. Indeed, the most specifically overrepresented pathway was glycolysis.

The comparison of upregulated proteins in P(+) and downregulated proteins in P(−) revealed only five proteins, with no significant results in the ontological analysis. 

A total of 137 proteins were commonly downregulated in both patients compared to the controls. From the analysis of biological processes, the top hierarchically enriched processes were platelet activation (FDR 1.07 × 10^−3^), endothelial cell migration (FDR 6.87 × 10^−5^), and the response to reactive oxygen species (FDR 1.44 × 10^−3^).

A total of 136 proteins were commonly upregulated in both patients compared to the controls. In the analysis of biological processes results, the top two groups were related to the production of molecular mediators of the immune response (FDR 3.45 × 10^−9^) and the carbohydrate derivative catabolic process (FDR 5.01 × 10^−3^).

A pathway enrichment analysis was performed on proteomic data to explore the pathways activated by the pathology. Pathways were explored using ClueGO 2.5.10 plugin against REACTOME_Pathways (89 version). The pathways obtained were filtered by a *p*-value cutoff of 0.05 (Benjamini–Hochberg correction) for statistical significance. Special attention was focused on those pathways containing differentially expressed proteins, and, notably, ‘Glucose metabolism’ (R-HSA:70326) and ‘Detoxification of Reactive Oxygen Species’ (R-HSA-3299685) emerged.

**Figure 3:**
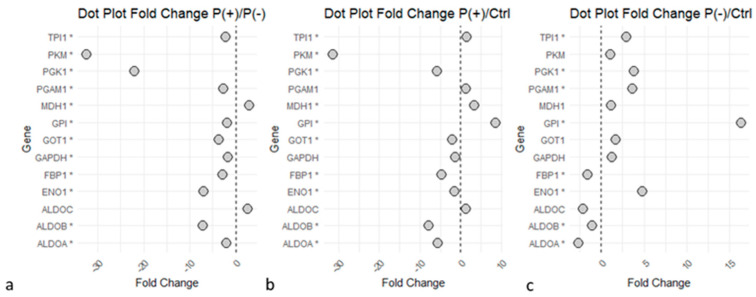
Dots plot of ‘Glycolysis’ and ‘Gluconeogenesis’ proteins. Graphs illustrate the proteins identified in the glucose metabolism pathways and their Fold Change (FC) values relative to the P(+)/P(−),P(+)/Ctrl, and P(−)/Ctrl ratios. Statistical significance is reached for *p*-values < 0.05 (*). (**a**) P(+)/P(−) ratio. (**b**) P(+)/Ctrl ratio. (**c**) P(−)/Ctrl ratio. Graphs indicate an upregulation of the enzymes in P(−) compared to P(+), several proteins downregulated in P(+)/Ctrl, while many proteins were upregulated in P(−)/Ctrl. The image was created using R (version 4.3.3, R Foundation for Statistical Computing, Vienna, Austria) software.

Within the ’Glucose metabolism’ pathway, numerous proteins were differentially expressed but showed a general opposite trend in the two patients (Figure 3a). To confirm the observations, the two ratios P(+)/Ctrl and P(−)/Ctrl between the two patients and healthy controls were also compared. It was observed that several proteins were downregulated in P(+), the patient with the more severe phenotype (Figure 3b), and upregulated in P(−), the patient with the less severe phenotype (Figure 3c).

**Figure 4:**
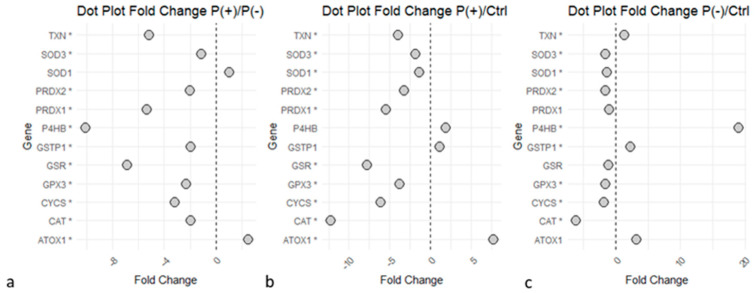
‘Detoxification of Reactive Oxygen Species’ proteins dysregulated in the disease. Statistical significance is reported as * for *p*-value < 0.05. (**a**) P(+)/P(−) ratio. (**b**) P(+)/Ctrl ratio. (**c**) P(−)/Ctrl ratio. Graphs indicate a downregulation of the enzymes in P(+) compared to P(−), and several proteins were downregulated in P(+)/Ctrl and P(−)/Ctrl. The image was created using R (version 4.3.3, R Foundation for Statistical Computing, Vienna, Austria) software.

The ‘Detoxification of Reactive Oxygen Species’ pathway revealed an impaired expression of proteins strictly linked to the antioxidant response in both patients compared to the controls (Figure 4), with the common increase of proteins such as protein disulfide-isomerase (P4HB), copper transport protein ATOX1 (ATOX1), and glutathione S-transferase P (GSTP1). Several proteins implied in the ROS synthesis and redox metabolism were commonly downregulated in the two patients, such as superoxide dismutase [Cu-Zn] (SOD1), catalase (CAT); key enzymes of glutathione (GSH) metabolism like glutathione reductase (GSR) and glutathione peroxidase 3 (GPX3); and proteins implicated in oxidative stress response, including the peroxiredoxin family (PRDXs). Despite the common deregulation in the two patients, several of these proteins showed a greater differential expression in the P(+)/Ctrl ratio compared to the P(−)/Ctrl ratio, as they also appeared to be less expressed in P(+) than in P(−).

The results also highlighted other proteins involved in the antioxidant activity, which showed an upregulation in the presence of the disease. Transketolase (TKT), an important mediator of the biosynthesis of the second antioxidant NADPH (Nicotinamide Adenine Dinucleotide Phosphate) [17], showed upregulation in both patients compared to the controls (FC 4.77 for P(−)/Ctrl and FC 2.63 for P(+)/Ctrl). Protein AMBP (AMBP), an antioxidant and tissue repair protein [18], also resulted in upregulation in both patients: (P(−)/Ctrl with a FC of 2.86, and in P(+)/Ctrl, a FC of 3.27).

## 3. Discussion

**Figure 5:**
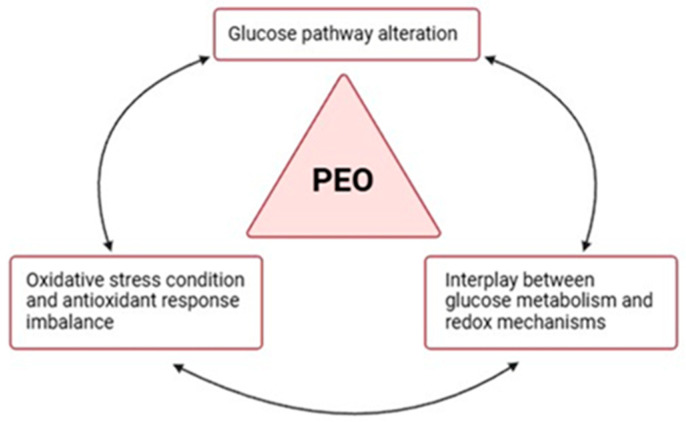
Dysregulation of the glycolysis–redox axis in PEO. Proteomic results support the hypothesis of an interrelation between glucose metabolism and redox mechanisms and their common imbalance in PEO mitochondrial disease. The image was created using BioRender (created in BioRender. Cicchinelli, M. (2022) BioRender.com/j97y594, accessed on 22 February 2024).

The decision to perform an in-depth proteomic analysis on urinary samples from two sisters affected by PEO, a rare neurogenetic disorder, arises from the clinical observation that subjects who share the same PEO pathogenic variant can present different phenotypic manifestations of the disease and a variable degree of clinical severity. The results obtained from our investigation suggest an imbalance of the glycolysis in the two mitochondrial patients enrolled in a redox axis, apparently related to different clinical severities (Figure 5). 

Glucose metabolism, with reference to glycolysis and gluconeogenesis, is the most differentiated process between the two patients, as highlighted by both biological process analysis and pathway enrichment. Specifically, observing the differentially expressed proteins implicated in these processes (Figure 3), most of them show a significant upregulation in the patient with the less severe phenotype, P(−). The same proteins exhibit an opposite trend in the clinically more severe patient, P(+), evidencing a downregulation in expression not only compared to patient P(−) but also when compared to the controls. 

Mitochondria are dynamic and multifunctional organelles with a central role in key physiological processes of a cell’s life [19]. Their most studied function is certainly the energy production in the ATP formation, achieved through the intricately regulated processes involving glucose metabolism and the OxPhos system. The strong interconnection and the dynamic interplay between these pathways have been well documented [20] and supported by numerous evidence, such as the activation of glycolysis following the inhibition of OxPhos [21,22], or the activation of mitochondrial function because of inhibited glycolysis [23,24]. 

Various triggers have a role in determining the transition between glycolysis and OxPhos, with a delicate balance depending on various conditions and stimuli [25]. Specifically, it has been observed in various human diseases that mitochondrial respiratory defects can initiate retrograde signaling pathways from this organelle to the nucleus [26,27], promoting changes in gene expression and in many metabolic processes, including glucose pathways.

Based on the metabolic dysregulation observed in the two patients, it is possible to hypothesize a correlation with their phenotypic severity. In addition, a connection between the different expression of glycolytic enzymes in the two patients and their clinical presentations could be assumed. In case of reduced mitochondrial functions, cells can increase ATP production through a metabolic shift toward glycolysis. Therefore, the overall increase in glycolytic activity in the less severely affected patient P(−) could potentially reflect a metabolic shift towards glycolysis to balance the ATP deficiency. 

It is also known that a severe and prolonged mitochondrial impairment can lead to a decrease in glycolytic flux due to the close co-dependence between glycolysis and the Krebs cycle [28], which is often significantly impacted by mitochondrial dysfunction [29]. Furthermore, the overproduction of NADH due to electron respiratory chain defects can disrupt the NADH/NAD+ ratio within cells, influencing the regulation of glycolytic enzymes [29]. These factors could explain the downregulation observed in P(+), and might indicate a failure of the mentioned compensatory mechanism due to more extensive mitochondrial damage or prolonged metabolic stress, comparable with the more pronounced clinical symptoms seen in P(+), such as greater muscle weakness and exercise intolerance. 

In addition to glucose metabolism, this study identified ROS detoxification as one of the key compromised processes in the pathological condition compared to the physiological state. ROS, normally generated as byproducts during metabolic redox reactions, actively participate in cell signaling events, influencing their structure and function [30]. Cellular ROS originate from various sources [31], with mitochondria playing a pivotal role through the synthesis of the most physiologically relevant ROS, superoxide anion radical (O2^·−^), and hydrogen peroxide (H_2_O_2_).

To avoid ROS imbalance, both mitochondria and cells have efficient antioxidant enzymes that play a crucial regulatory role against the majority of oxidants formed in physiological conditions [31]. When ROS production, stimulated by pathological stressors, escapes the control of the antioxidant system, an alteration of the biological redox signaling occurs, leading to mitochondrial and cellular damage [30]. This redox system imbalance is defined ‘oxidative stress’ and is a characteristic condition found in various pathological states and documented in PMM [32].

Altered ROS levels, as a consequence of the PMM variants, may damage the mitochondrial genome, intensifying respiratory defects and increasing the production of other ROS and lipid peroxides [33]. Due to this evidence, ROS overproduction and the presence of an oxidative stress condition are considered potential pathomechanisms for mitochondrial diseases [34].

Based on the results of the enrichment analyses, several proteins associated with ROS metabolism and elimination show strong dysregulation in the pathological state. Specifically, proteins such as SOD1 and CAT, involved in the transformation of superoxide (O_2_^−^) and hydrogen peroxide (H_2_O_2_), exhibit downregulation in the patients [35]. Both patients also show downregulation of glutathione metabolism enzymes, including GSR and GPX3, crucial effectors of the antioxidant response [35,36]. The expression of the peroxiredoxin family (PRDXs), involved in oxidative stress response, is also downregulated in the patients. Overall, the enzymes involved in cellular redox balance show a deficiency in expression in the pathological state, with a more significant downregulation in the severe patient P(+). At the same time, other enzymes with known antioxidant activity show upregulation in the pathological state, in particular, TKT, a key enzyme in NADPH production through the pentose phosphate pathway (PPP). NADPH produced by TKT is essential in glutathione metabolism and thus plays an important role in antioxidant activity.

In observing the results, it is evident that P(+) shows greater impairment of the described mechanisms, highlighted by the downregulation of key enzymes involved in ROS metabolism. On the other hand, P(−) also shows clear impairment of the same enzymes, but with a smaller delta compared to the control condition. Even in this case, it is possible to hypothesize that the different degrees of impairment could correlate with their clinical presentations. 

**Figure 6:**
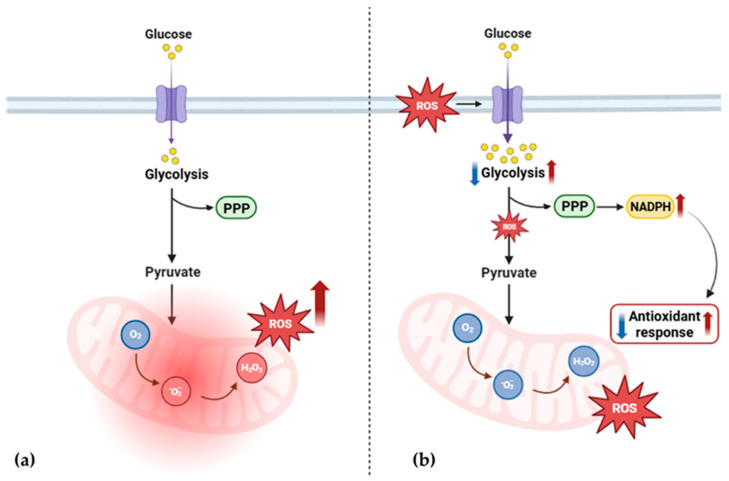
Proposed alteration of ROS and glucose metabolism in PEO mitochondrial disease. (**a**) Mitochondrial dysfunction triggers increased ROS production and leads to oxidative stress. (**b**) In response, glycolytic flux is altered [34,35,36], further impairing the production of NADPH and the antioxidant response. PPP, pentose phosphate pathway; ROS, reactive oxygen species; NADPH, nicotinamide adenine dinucleotide phosphate; O_2_, oxygen; O_2_^−^, superoxide anion radical; H_2_O_2_, hydrogen peroxide. The image was created using BioRender (created in BioRender. Cicchinelli, M. (2022) BioRender.com/y52u291, accessed on 22 February 2024).

The interplay between ROS and glucose metabolism [31,37,38,39] plays a crucial role in diseases involving OxPhos dysfunction. It has been observed that the increase in ROS production and changes in glycolytic pathway expression co-occur in various disorders characterized by OxPhos dysfunctions. As Liemburg-Apers et al. elegantly suggested [40], excessive ROS production could stimulate glucose uptake, affecting both glycolysis and gluconeogenesis. This response likely serves as part of the oxidative stress response, as glucose may help counteract oxidative damage. Additionally, this increase in glucose uptake affects the pentose phosphate pathway, enhancing NADPH production, which regulates glutathione metabolism and influences the antioxidant defense system [31,41]. The proteomic data from our study support this relationship. Both patients display dysregulation in ROS metabolism and a significant impairment in glycolytic enzymes. This suggests the presence of a cause–effect cycle in which mitochondrial dysfunction triggers increased ROS production, leading to oxidative stress (Figure 6a). In response, glycolytic flux is altered, further impairing the antioxidant response (Figure 6b). 

However, differences in enzyme expression suggest multiple patterns of metabolic adaptation. This variation in response may contribute to inter-individual differences observed in clinical symptoms and may be related, at least in part, to the phenotypic heterogeneity commonly observed in mitochondrial diseases [42,43].

## 4. Patients and Methods

### 4.1. Patients

Urine samples from the patients and controls were collected and stored at the Fondazione Policlinico Universitario Agostino Gemelli IRCCS, Rome, Italy. The two subjects were identified within the cohort of patients with mitochondrial disease followed at our center for the intriguing combination of clinical variability and identical genetic variants. All patients underwent a clinical evaluation by a neurology expert in mitochondrial medicine and were classified according to the clinical phenotypes and based on the molecular characterization. The disease burden was assessed by the Newcastle Mitochondrial Disease Adult Scale (NMDAS), assigning a different severity score between the two patients [16]. The patient with the most severe clinical phenotype is indicated as P(+), and the one with a mild phenotype as P(−). The patients did not follow any special diets, habits, or physical activity. Urine collection was performed after 8 h of fasting to minimize any type of variable. Control samples were collected from healthy volunteers without any kind of disease.

### 4.2. Urine Samples Collection

Mid-stream of the second morning’s urination was obtained from the patients and healthy controls and collected in sterile urine cup. Urine samples were centrifuged at 3000 g (4 °C) for 30 min to remove cells and debris. Then, each supernatant was stored in aliquots of 10 mL at −80 °C. The sample collection method followed the Standard Protocol for Urine Collection and Storage guideline, applicable for the analysis of urine proteins, described and recommended by the Human Kidney and Urine Proteome Project (HKUPP).

### 4.3. Urine Samples Preparation

The samples were thawed at room temperature and subjected to a dialysis procedure for the elimination of salts and interferents, employing the dialysis tubing, and were benzoylated—avg. flat width 32mm (Sigma-Aldrich, Saint Louis, MO, USA).

The dialysis tubes were prepared in accordance with the protocol established by Sigma-Aldrich: sulfur compounds were removed by treating with a 0.3% (*w*/*v*) sodium sulfide solution at 70 °C for one minute, washing with hot water (60 °C) for two minutes, followed by acidification with 0.2% sulfuric acid and a second wash with hot water to remove the acid. Each urine aliquot was transferred to a pretreated dialysis tube, which was then submerged in a 10 mM PBS solution, ensuring a 1:100 ratio between the sample volume and the buffer. The tubes were securely sealed, and overnight dialysis was carried out at room temperature. The dialyzed samples were collected and concentrated using Vivaspin^®^ 6, 5000 MWCO PES, 100pc (Sartorius Corporation, Bohemia, NY, USA) at 3000 g (room temperature) to achieve a final volume of 500 µL. In each sample, PIC (at a 1:100 ratio) was added, followed by drying in a speed vac. Each dried urine sample was dissolved in Urea Buffer solution (8 M urea and 100 mM Tris).

The protein concentration was determined through the Bradford Protein Assay (Bio-Rad Laboratories, Hercules, CA, USA).

### 4.4. Enzymatic Digestion and Mass Spectrometry Analysis

Protein digestion was performed according to the filter-aided sample preparation (FASP) protocol that combines both purification and digestion [44,45] of the proteins. Briefly, 50 µg of proteins from each sample were reduced (DTT 8 mM in urea buffer −8 M urea and 100 mM Tris), alkylated (IAA 50 mM in urea buffer −8 M urea and 100 mM Tris), and digested by trypsin on filter tubes Microcon^®^ Centrifugal Filter Devices (Merck Millipore Ltd., Cork, Ireland) at a final concentration of 1 μg/μL.

Bottom-up proteomic analysis was performed by UltiMate™ 3000RSLCnano—HPLC System (Thermo Fisher Scientific, Waltham, MA, USA) coupled to a high-resolution Orbitrap Fusion Lumos Tribrid Mass Spectrometer (Thermo Fisher Scientific) with an ESI source. Peptides were separated via a PepMap RSLC C18 column 2µM, 100Å, 50 µm × 15 cm (Thermo Fisher Scientific) in gradient elution, using an aqueous solution of FA (0.1%, *v*/*v*) as eluent A, and ACN/water (80:20, *v*/*v*) with 0.1% (*v*/*v*) FA as eluent B. The following step gradient was applied (run time 155 min): 3% eluent B and 97% eluent A (min 0–110), 20% eluent B and 80% eluent A (min 110–120), 40% eluent B and 60% eluent A (min 120–125), 90% eluent B and 10% eluent A (min 125–145), 3% eluent B and 97% eluent A (min 145–155), (% values, *v*/*v*) at a flow rate of 0.300 μL/min. The injection volume was 5 μL (1 μg of peptides), ion source type NSI, positive polarity (voltage 1800V), ion transfer tube temp. 275 °C. The following MS parameters were set: the acquisition of high-resolution MS/MS spectra was carried out in data-dependent scan mode (DDS) with Orbitrap as detector, a resolution of 120.000 in 375–1500 m/z range of acquisition and higher-energy collisional dissociation (HCD) fragmentation. Samples were analyzed in triplicate.

### 4.5. MS Data Analysis and LFQ Quantification

The bottom-up MS/MS data were elaborated using Proteome Discoverer 2.4.1.15 (Thermo Fisher Scientific) based on the SEQUEST HT algorithm (University of Washington, USA, licensed to Thermo Electron Corp., San Jose, CA, USA) against UniProtKB/Swiss-Prot *homo sapiens* proteome database. The setting parameters were as follows: minimum precursor mass 350 Da; maximum precursor mass 5000 Da; total intensity threshold 0.0; minimum peak count 1; signal-to-noise (S/N) threshold 1.5; precursor mass tolerance 10 ppm; fragment mass tolerance 0.02 Da; use average precursor mass false; use average fragment mass false; maximum missed cleavage 2; minimum peptide length and maximum peptide length 6 and 144, respectively; oxidation/+15.995 Da (M) as dynamic modification; carbamidomethyl/+57.021 Da (C) as static modification; FDR rate at 0.01 (strict) and 0.05 (relaxed). Protein abundance was obtained through LFQ analysis with the following settings: precursor abundance area, protein abundance calculation top 3 average.

### 4.6. Bioinformatic Analysis

The results were filtered in high confidence for ≥2 unique peptides. Triplicate mean abundance was considered for bioinformatics analysis.

Fold Change (FC) ratios and *p*-values were calculated using Matlab Statistics (9.13 version) and Machine Learning Toolbox and Bioinformatics Toolbox (https://it.mathworks.com/products/statistics.html; https://it.mathworks.com/products/bioinfo.html, accessed on 22 February 2024; R2022b version, MathWorks Inc., Natick, MA, USA). The pathway analysis was performed using Reactome (89 version; http://reactome.org/, accessed on 6 September 2024) database and ClueGO (2.5.10 version, Cytoscape Consortium) plugin for Cytoscape (3.10.1 version, Cytoscape Consortium). The ontological analysis was performed using Panther Protein (16.0 version; www.pantherdb.org, accessed on 6 September 2024).

## 5. Conclusions

This study provides insights into the molecular mechanisms underlying phenotypic variability in patients affected by PEO, highlighting the impairment of glucose metabolism and ROS detoxification as potential contributors to clinical severity. Through a urinary proteomic analysis, key differences in the expression of proteins involved in these pathways were identified in two sisters affected by PEO associated with the c.1075G>A variant in the *TWNK* gene, but presenting a distinct clinical severity. 

The most differentiated process between the patients was glucose metabolism, particularly glycolysis and gluconeogenesis. In the patient with the less severe phenotype, P(−), we observed an upregulation of several glycolytic enzymes, suggesting a compensatory shift towards glycolysis to maintain ATP production despite the mitochondrial dysfunction. This potential adaptive mechanism might partially compensate for the loss of oxidative phosphorylation (OxPhos) efficiency. Conversely, the more severely affected patient, P(+), exhibited significant downregulation of glycolytic enzymes, indicating a failure of this assumed compensatory mechanism, or the presence of more extensive mitochondrial damage.

In addition to glucose metabolism, ROS detoxification pathways were also significantly impaired in both patients. The downregulation of key antioxidant enzymes, coupled with a deficiency in glutathione metabolism enzymes, might end in an oxidative stress condition and mitochondrial damage. The patient with the more severe phenotype exhibited a greater degree of dysregulation in these antioxidant systems, supporting the hypothesis that insufficient ROS detoxification could contribute to more severe clinical outcomes in this specific pathological condition.

Interestingly, despite the downregulation of several antioxidant enzymes, an upregulation of TKT, a key enzyme in NADPH production via the pentose phosphate pathway, was observed in both patients. NADPH is crucial for maintaining the redox balance and its upregulation may represent an effort by the cells to correct the oxidative stress condition. 

The interplay between ROS and glucose metabolism was also considered, hypothesizing that, when impaired, it creates a potential cause–effect loop that may reflect the clinical severity. 

This study has limitations, including the lack of repeated proteomic analyses at different time points and the small number of patients enrolled. For the reasons mentioned above, the reported data need to be validated by longitudinal investigations in a large number of patients, also combining different omics technologies (metabolomics, transcriptomics, genomics, including epigenetics).

In conclusion, the differential expression of key enzymes in both glycolysis and ROS detoxification pathways in the two sisters suggests a complex relationship between mitochondrial dysfunction, its metabolic effects, and clinical severity in PEO. Based on urinary proteomic data, it is possible to hypothesize that the phenotypic variability between patients with the same genetic variant may be justified by the ability of biochemical adaptation to the underlying mitochondrial dysfunction.

## Data Availability

The data that support the findings of this study are available from the corresponding author, upon reasonable request.
